# e²FAST: Integrating Inferior Vena Cava Ultrasound Into the Extended Focused Assessment With Sonography for Trauma as a Rapid Diagnostic Tool of Hemorrhagic Shock in Trauma Patients

**DOI:** 10.7759/cureus.98529

**Published:** 2025-12-05

**Authors:** Fawziah Alsalmi, Joe Nemeth, Mahmood Alshaaban

**Affiliations:** 1 Emergency Medicine, King Fahad Armed Forces Hospital, Jeddah, SAU; 2 Emergency Medicine/Trauma, McGill University, Montreal, CAN; 3 Emergency Medicine/Trauma, King Hamad University Hospital, Muharraq, BHR

**Keywords:** e-fast, efast protocol, emergency medicine, focused assessment with sonography in trauma (fast), hemorrhagic shock, inferior vena cava ultrasound (ivc-us), massive hemorrhage protocol, point-of-care ultrasound (pocus), resuscitation, trauma

## Abstract

Hemorrhagic shock remains a leading cause of preventable trauma-related deaths, highlighting the need for rapid and accurate diagnosis. This study evaluates whether integrating inferior vena cava ultrasound (IVC-US) into the extended focused assessment with Sonography for trauma (eFAST), referred to as “e²FAST,” improves early shock detection and guides fluid resuscitation in trauma patients. A comprehensive narrative review was conducted to assess the diagnostic and prognostic value of IVC-US as a rapid diagnostic tool for hemorrhagic shock in trauma patients. Studies published from 2000 to January 2025 were searched across PubMed, MEDLINE, EMBASE, Cochrane Library, and Scopus databases. Studies were included if they involved adult trauma patients and evaluated IVC diameter or the collapsibility index (IVC-CI) to determine volume status, shock, or fluid responsiveness. Two independent reviewers screened and selected studies based on predefined inclusion criteria. In total, 13 eligible studies, including randomized controlled trials, prospective and retrospective cohorts, and cross-sectional analyses, were reviewed qualitatively. Key outcomes extracted included diagnostic accuracy, fluid responsiveness, transfusion requirements, intensive care unit (ICU) admission, and mortality. IVC-US demonstrated a sensitivity of 71% and specificity of 75% in detecting hypovolemia. A smaller IVC diameter (<1.5 cm) was significantly associated with increased ICU admissions (51.3%), higher transfusion rates (12.2%), increased emergency surgery requirements (16.2%), and elevated mortality (13.5%). Additionally, an IVC-CI greater than 38.5% provided 80% sensitivity and 85.7% specificity for predicting fluid responsiveness in patients with major blunt trauma. Post-resuscitation assessments (IVC-CI ≤28.6%) were predictive of fluid unresponsiveness (80% sensitivity, 75% specificity). Moreover, IVC-US reliably predicted 24-hour fluid requirements, helping in real-time fluid management. Integrating IVC-US into the eFAST as the e²FAST protocol enhances diagnostic accuracy for identifying hemorrhagic shock and provides critical real-time guidance for fluid resuscitation. Given its non-invasive nature, reliability, and predictive value for patient outcomes, standardized inclusion of IVC measurements in trauma protocols is recommended. Future research should prioritize multicenter randomized trials and standardized operator training to minimize variability and support broad clinical implementation.

## Introduction and background

Hemorrhagic shock remains a leading cause of preventable death in trauma patients, necessitating early detection and prompt intervention [1]. While the focused assessment with sonography for trauma (FAST) and extended focused assessment with sonography for trauma (eFAST) examinations facilitate the detection of hemoperitoneum and other life-threatening conditions [2], neither includes static or dynamic assessment of the inferior vena cava (IVC) [3]. Point-of-care ultrasound (POCUS) is an invaluable, non-invasive tool in trauma care, offering a potential tool for real-time evaluation of a patient’s physiological status [4]. Specifically, measurements of IVC diameter and the IVC collapsibility index (IVC-CI), which quantifies the percentage change in IVC diameter with respiration, can provide essential information about fluid responsiveness and hemodynamic status [5]. This review evaluates the proposal of integrating IVC ultrasound (IVC-US) assessment into the eFAST protocol to enhance early detection of hypovolemia and resuscitation strategies, ultimately optimizing the detection of early shock and guiding volume resuscitation in a more real-time manner.

## Review

Methodology

Study Design

This study was designed as a structured narrative review to assess the diagnostic and prognostic value of IVC-US in evaluating hemorrhagic shock in trauma patients. Additionally, it aims to explore how IVC-US can be integrated into eFAST, proposing an improved diagnostic framework called e²FAST. A narrative review approach was intentionally selected due to the heterogeneity of available studies in terms of design, patient populations, measurement techniques, and reported outcomes, which made a systematic review or meta-analysis unfeasible. More importantly, this approach was deemed suitable for introducing and contextualizing the e²FAST protocol as an innovative extension of eFAST, emphasizing the role of IVC-US as a rapid, non-invasive tool for the early detection of hemorrhagic shock in trauma assessments. As this study involved secondary analysis of previously published literature, no human subjects were directly involved; therefore, Institutional Review Board approval was not required.

Inclusion and Exclusion Criteria

Studies were included if they (1) focused on the use of IVC-US in critically ill, spontaneously breathing adult trauma patients; (2) assessed volume status, shock, or fluid responsiveness using IVC diameter or IVC-CI; (3) were published in English from 2000 to January 2025. Studies were excluded if they (1) involved pediatric or non-trauma groups; (2) lacked sufficient data or a clear methodology; (3) did not specifically evaluate ultrasound for volume or shock assessment.

Both observational and interventional study designs were considered, including randomized controlled trials (RCTs), cohort studies, and pertinent reviews.

Search Strategy

A thorough search was performed across five major databases, i.e., PubMed, MEDLINE, EMBASE, Cochrane Library, and Scopus. The search included publications from January 2000 to January 2025 using various combinations of the following terms: “Inferior Vena Cava,” “IVC,” “ultrasound,” “trauma,” “hemorrhagic shock,” “shock,” “hypotension,” “fluid status,” “volume assessment,” “FAST,” “eFAST,” and “point-of-care ultrasound (POCUS).” To minimize selection bias and enhance comprehensiveness, broad search terms and multiple database sources were utilized, and studies of various designs (RCTs, cohort studies, and cross-sectional analyses) were included. The reference lists of key studies were manually reviewed to ensure completeness. Two independent reviewers screened titles, abstracts, and full texts based on the predefined inclusion criteria. Disagreements were resolved through discussion and consensus to reduce bias.

Data Extraction

Data obtained from each of the 13 eligible studies included: (1) study characteristics: design, population, and sample size; (2) ultrasound techniques: mode (B-mode or M-mode) and measurement site; (3) IVC parameters: diameter and IVC-CI; (4) clinical outcomes: diagnostic accuracy, fluid responsiveness, transfusion requirements, intensive care unit (ICU) admission, and mortality; (5) study limitations reported by the authors.

Sample Size Consideration

No formal sample size calculation was applicable, as this review analyzed published literature. The included studies involved sample sizes ranging from 50 to 350 trauma patients, representing diverse trauma settings and severities.

Analysis

A qualitative synthesis was performed to assess the effectiveness of IVC-US in trauma settings. The analysis concentrated on the diagnostic accuracy of IVC diameter and IVC-CI, predictive value for fluid responsiveness and transfusion needs, and impact on clinical outcomes, such as ICU admission and mortality. Findings were summarized to emphasize the diagnostic reliability and prognostic potential of IVC-US and to support its proposed addition to the e²FAST protocol for real-time trauma resuscitation.

Results

This review included studies with different designs, including RCTs, prospective and retrospective cohort studies, and cross-sectional studies. The sample sizes ranged from 50 to 350 patients, and the research was conducted across multiple trauma centers.

Diagnostic Accuracy

The studies consistently show that IVC-US is valuable for assessing fluid status, diagnosing shock, and guiding resuscitation efforts. Research findings indicate that the IVC diameter has a sensitivity of 71% and a specificity of 75% [6]. It has proven to be a reliable, non-invasive method for assessing intravascular volume status in trauma patients with hemorrhagic shock [7,8].

Prognostic Indicators

Several studies have highlighted the prognostic value of IVC diameter in predicting outcomes among trauma patients. Notably, an IVC diameter of less than 1.5 cm has been identified as a predictor of poor prognosis [7]. Patients with a small IVC diameter had higher rates of ICU admission (51.3%), increased blood transfusion needs (12.2%), emergency surgery (16.2%), and higher mortality (13.5%) [7]. Similarly, a smaller IVC diameter (equal to or smaller than 9 mm) is strongly correlated with shock in trauma patients [9]. Conversely, an IVC diameter of more than 13 mm has been associated with a reduced need for massive transfusion in blunt trauma patients [10].

Fluid Responsiveness

Assessing the IVC diameter and IVC-CI is valuable for predicting fluid responsiveness and outcomes in trauma patients [11]. In cases of major blunt trauma, an IVC-CI greater than 38.5% upon arrival was linked to 80% sensitivity and 85.7% specificity for predicting fluid responsiveness [8]. After one hour of resuscitation, an IVC-CI of 28.6% or less showed 80% sensitivity and 75% specificity for predicting fluid unresponsiveness [8].

Clinical Outcomes

Beyond diagnostic and predictive accuracy, IVC-US measurements have demonstrated strong correlations with important clinical outcomes. IVC-US measurements have been shown to predict 24-hour fluid requirements in trauma patients [11], indicating that IVC-US can effectively guide fluid management during the early stages of trauma resuscitation. Overall, the evidence supports adding IVC-US into trauma assessment protocols as an early diagnostic and prognostic adjunct. Table 1 summarizes the main findings from the reviewed studies, including diagnostic accuracy, prognostic thresholds, and clinical implications.

**Table 1:** Summary of the key findings. ICU = intensive care unit; IVC = inferior vena cava; IVC-CI = inferior vena cava collapsibility index

Parameter	Finding	Diagnostic accuracy	Outcome	Reference
IVC diameter as a diagnostic tool	IVC diameter used to detect hypovolemia	Sensitivity: 71%, specificity: 75%	Effective for identifying hypovolemia and guiding early resuscitation	[6]
Predictive value of IVC diameter	IVC diameter <1.5 cm is associated with poor prognosis	-	Increased ICU admissions (51.3%), higher transfusion rates (12.2%), emergency surgery (16.2%), and mortality (13.5%)	[7]
IVC-CI	IVC-CI >38.5% indicates fluid responsiveness	Sensitivity: 80%, specificity: 85.7%	Predicts fluid responsiveness in major blunt trauma	[8]
Resuscitation prediction	Upon arrival, the IVC ultrasound estimates of 24-hour fluid requirement	-	Accurately predicts resuscitation needs during acute trauma management	[10]

Discussion

Clinical Rationale for e²FAST

Hemorrhagic shock remains the leading cause of preventable death in trauma, and survival depends on early recognition, prompt resuscitation, hemorrhage control, definitive hemostasis, and achieving resuscitation endpoints [12]. Ultrasound has progressively expanded its role in trauma evaluation and resuscitation. The FAST examination, initially designed to identify hemopericardium and hemoperitoneum, has become a cornerstone of the initial trauma survey. Over time, this protocol has developed into eFAST, which adds thoracic views to detect pneumothorax and hemothorax, thereby broadening its diagnostic capabilities [2]. Beyond these established uses, ultrasound also allows rapid, non-invasive assessment of the IVC, providing estimates of intravascular volume status. Including IVC-US in trauma assessment helps clinicians detect occult hypovolemia and adds a physiological perspective to the primarily anatomical evaluation of eFAST [13]. Unlike traditional perfusion markers such as vital signs, urine output, or serum lactate, static measures such as central venous pressure (CVP) and pulmonary capillary wedge pressure (PCWP) have been used to estimate intravascular volume and cardiac preload. However, these measures are limited by their procedural complexity and potential risks. Conversely, IVC-US offers dynamic, real-time hemodynamic monitoring [14,15]. This capability addresses a long-standing gap in trauma care, enabling timely interventions that may prevent progression to irreversible hemodynamic collapse.

Diagnostic Accuracy and Prognostic Value

A small IVC diameter has consistently been linked with poor prognosis in trauma patients, associated with higher transfusion needs, increased ICU admissions, and higher mortality rates [14,16]. Hypotensive trauma patients often show significantly smaller IVC diameters compared to normotensive patients, highlighting the ability of IVC-US to detect hypovolemia even when vital signs appear normal [16]. Both IVC diameter and IVC-CI accurately reflect blood volume and perfusion status, strongly correlating with CVP [17]. A reduced collapsibility indicates elevated CVP, while a flat, non-distensible IVC is strongly linked to shock markers, including hypotension, increased lactate, and poor outcomes [16,17]. Beyond initial detection, IVC-US also provides important prognostic insights. A smaller IVC diameter before resuscitation has been associated with shock indicators such as lower blood pressure and higher lactate levels [18]. In blunt trauma, initial IVC diameter helps forecast the need for massive transfusions, while serial IVC measurements taken one hour after resuscitation can estimate fluid requirements for the next 24 hours, offering real-time feedback on resuscitation progress [8,10]. The prognostic importance of IVC assessment was further highlighted by Chien et al., who showed that an admission IVC volume of ≤18 mL, calculated from cross-sectional area, was an independent predictor of massive transfusion, even after accounting for injury severity and other clinical factors (adjusted odds ratio = 4.17, p < 0.01) [19]. This measurement was also associated with severe shock physiology, such as hypotension, tachycardia, and increased lactate levels. Overall, these results support the use of IVC-US as both a diagnostic and prognostic tool, helping to identify high-risk trauma patients, guide activation of massive transfusion protocols, and tailor resuscitation strategies within the proposed e²FAST framework.

Comparison With Traditional Modalities

Traditional methods for assessing intravascular volume and perfusion in trauma, such as vital signs, urine output, serum lactate, and static pressure indicators, such as CVP or PCWP, often lack sensitivity and specificity for detecting early hypovolemia [14,16]. Vital signs may remain deceptively normal due to compensatory mechanisms, while urine output and lactate are delayed markers that usually indicate shock only after significant physiological compromise [17]. Similarly, invasive pressure monitoring through CVP or PCWP has consistently been shown to correlate poorly with actual intravascular volume and provides limited predictive value for fluid responsiveness [18]. In contrast, IVC-US offers a rapid, repeatable, and noninvasive bedside method for assessing intravascular volume status. Studies demonstrate that both IVC diameter and collapsibility reliably correlate with CVP and fluid responsiveness [20]. Unlike traditional markers, IVC-US provides real-time dynamic hemodynamic monitoring, enabling clinicians to detect occult hypovolemia earlier and customize resuscitation more precisely [21]. When integrated into eFAST as the proposed e²FAST protocol, IVC assessment expands the examination from mainly an anatomical review to a combined anatomical and physiological evaluation, improving diagnostic accuracy and clinical decision-making in trauma resuscitation.

Technical Considerations

Understanding the best way to measure the IVC is crucial for integrating it effectively into trauma assessment. Static measurements are easier to perform and especially useful in borderline cases where clinical signs are unclear [20,21]. However, they depend heavily on the operator and can be affected by patient-specific factors such as obesity, right heart dysfunction, lung hyperinflation, or increased intra-abdominal pressure [22]. Dynamic assessments, such as IVC-CI, provide additional information, and combining static and dynamic parameters yields the most accurate representation of intravascular volume status [23]. These technical details align with broader trauma ultrasound frameworks, including the Rapid Ultrasound in Shock and Hypotension (RUSH) examination. Measuring the IVC diameter approximately 2 cm below the right atrial junction correlates strongly with CVP in spontaneously breathing patients [24]. Thresholds such as an IVC diameter less than 1.5 cm with collapsibility greater than 50% reliably indicate low CVP, while diameters over 2.5 cm with minimal collapsibility suggest high CVP [24]. In hypovolemic shock, typical sonographic signs include a flat, collapsible IVC accompanied by FAST-positive free fluid; in obstructive shock, the IVC appears distended and non-collapsible, sometimes with absent lung sliding on eFAST [24]. Applying these interpretive patterns using the proposed e²FAST protocol enables clinicians to detect hemorrhage and differentiate its hemodynamic effects from those of other shock states in real time.

Integration Into Trauma Protocols

The eFAST examination is widely recognized as a cornerstone of trauma assessment, enabling quick detection of life-threatening issues such as hemoperitoneum, hemopericardium, pneumothorax, and hemothorax [2,3]. The proposed addition of IVC-US to this framework, forming the e²FAST protocol, is both feasible and practical, as it does not significantly increase the exam duration [7,9]. The integration feels natural because the subxiphoid view, already part of the eFAST protocol, allows easy visualization of the IVC. The subxiphoid transabdominal long-axis view, measured about 2 cm below the junction of the hepatic vein and the IVC, is considered the most reliable method for assessing IVC diameter due to its high intra-class correlation coefficient [24]. Adding this measurement to the routine eFAST creates a seamless workflow that allows both structural and functional assessments to occur simultaneously. Importantly, adding IVC assessment to eFAST helps distinguish different shock states. A flat, collapsible IVC indicates hypovolemic shock, while a distended, non-collapsible IVC may suggest obstructive causes such as cardiac tamponade or tension pneumothorax [24]. This functional data, combined with anatomical findings, enhances clinical decision-making and guides more accurate resuscitation strategies. Therefore, incorporating IVC-US into trauma protocols can significantly improve patient outcomes while maintaining the speed and practicality needed in emergency trauma care [7,9].

Proposed Standardized Protocol: e²FAST

**Figure 1:**
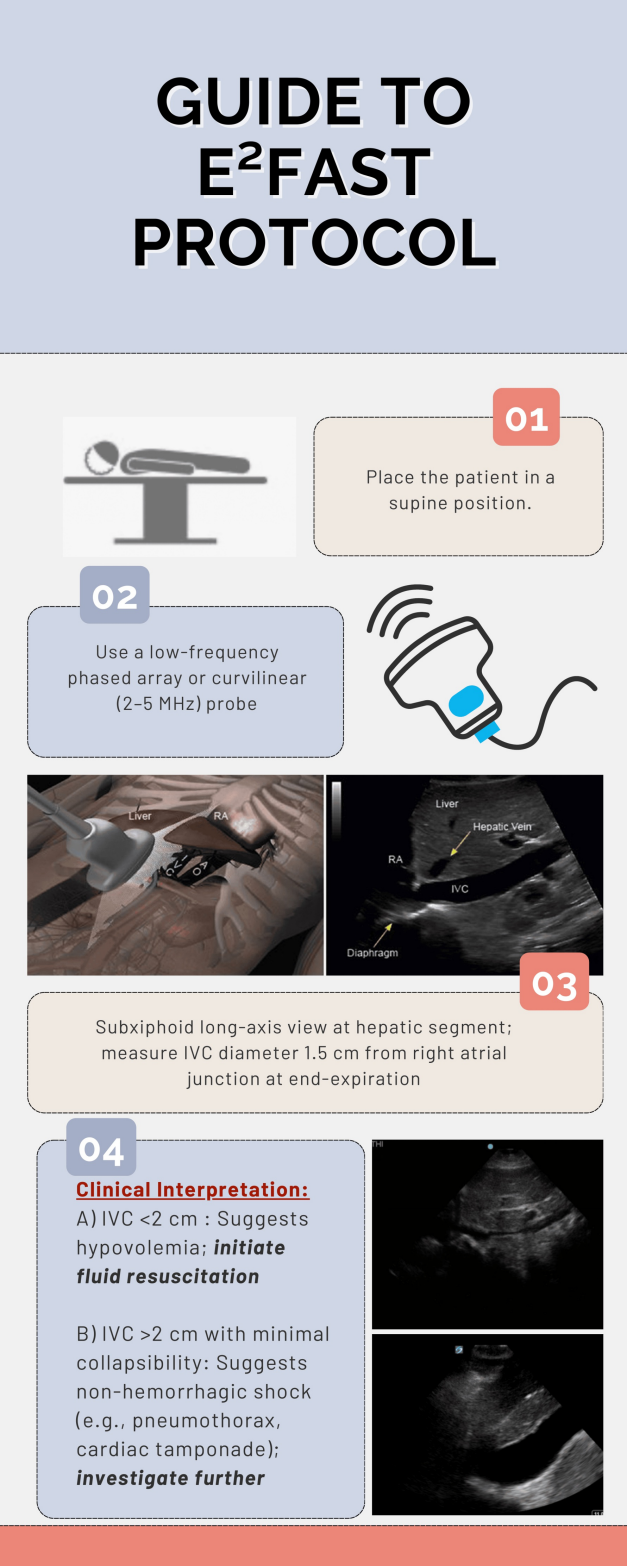
Proposed standardized protocol of e²FAST. Adapted from “The ICU Ultrasound Pocket Book,” Killu et al. [25]. The main author granted permission. IVC = inferior vena cava; RA = right atrium

In the proposed e²FAST protocol, the patient is positioned supine, and a low-frequency phased array or curvilinear transducer (2-5 MHz) is used. During the Subxiphoid view of the eFAST examination, the IVC is evaluated by measuring its diameter 2 cm below the hepatic vein-IVC junction. The transducer is placed just below the xiphoid process, angled cephalad toward the heart, then tilted caudally to visualize the long axis of the IVC as it enters the right atrium, which serves as a key anatomical landmark. As shown in Figure 1, this simple addition enhances the standard eFAST by providing real-time hemodynamic assessment and supports more accurate fluid resuscitation in critically injured patients.

Limitations

The reviewed studies have several key limitations that impact the strength and generalizability of the available evidence. Many studies relied on retrospective or observational designs, often conducted at a single center, which limit their ability to establish causation and reduce external validity. Operator dependency remains a major confounder, as the accuracy of IVC-US measurements heavily depends on the clinician’s skill and experience. Variability in training and expertise among personnel may have contributed to inconsistencies in study results and outcomes. Selection bias was also present in some studies where expert consensus guided diagnoses or where treatment decisions were influenced by ultrasound findings, potentially skewing outcome measures. Additionally, most studies focused on immediate or short-term endpoints such as resuscitation markers, transfusion needs, or early mortality, without examining longer-term outcomes such as survival, functional recovery, or cost-effectiveness. Lastly, the rapid evolution of ultrasound technology and emerging adjuncts means that many published studies may not reflect the most current capabilities of POCUS. Overall, these limitations highlight the need for larger, prospective, multicenter studies to validate the integration of IVC-US into trauma care.

Future directions

Future research should focus on multicenter RCTs to confirm the diagnostic and prognostic accuracy of IVC-US and to evaluate the clinical benefits of incorporating IVC-US into eFAST as part of the e²FAST protocol. At the same time, implementing standardized training and credentialing programs is essential to reduce operator-dependent variability and ensure consistent image acquisition and interpretation across providers. Advances in technology offer new opportunities. The integration of artificial intelligence and decision-support tools could automate IVC measurement and interpretation, decreasing subjectivity and boosting efficiency in high-acuity trauma scenarios. Moreover, expanding the use of e²FAST into prehospital and resource-limited settings, including military and disaster medicine, could facilitate earlier detection of hemorrhagic shock and enable more prompt resuscitation. Finally, endorsement of e²FAST by professional trauma and emergency medicine societies and its inclusion in standardized trauma care protocols would encourage broader adoption. These steps, along with strong clinical validation, could turn IVC-US from an innovative tool into a widely accepted standard of care in trauma resuscitation.

## Conclusions

This review emphasizes the clinical utility of IVC-US as a rapid, non-invasive tool for evaluating intravascular volume status and guiding resuscitation in trauma patients, especially those with hemorrhagic shock. Evidence shows that IVC diameter and IVC-CI are reliable indicators of hypovolemia and fluid responsiveness, with clear thresholds that support timely and accurate clinical decisions. Incorporating IVC assessment into eFAST improves diagnostic accuracy by adding hemodynamic evaluation to the standard detection of free fluid. The use of IVC-US is particularly valuable in borderline or early hemorrhagic shock cases, where clinical signs can be subtle. Standardized application of the proposed e²FAST protocol may enhance trauma care by allowing real-time, physiologically guided resuscitation. Additionally, targeted operator training and protocol standardization are crucial to ensuring reliability, reproducibility, and better patient outcomes. Future research should focus on multicenter RCTs to validate these findings and promote wider clinical adoption of e²FAST.
